# Beyond Bernoulli: Improving the Accuracy and Precision of Noninvasive Estimation of Peak Pressure Drops

**DOI:** 10.1161/CIRCIMAGING.116.005207

**Published:** 2017-01-16

**Authors:** Fabrizio Donati, Saul Myerson, Malenka M. Bissell, Nicolas P. Smith, Stefan Neubauer, Mark J. Monaghan, David A. Nordsletten, Pablo Lamata

**Affiliations:** From the King’s College London, Division of Biomedical Engineering and Imaging Sciences, St. Thomas’ Hospital, The Rayne Institute, United Kingdom (F.D., N.P.S., D.A.N., P.L.); Division of Cardiovascular Medicine, Radcliffe Department of Medicine, University of Oxford, United Kingdom (S.M., M.M.B., S.N.); University of Auckland, New Zealand (N.P.S.); and Department of Non Invasive Cardiology, King’s College Hospital, London, United Kingdom (M.J.M.).

**Keywords:** Bernoulli principle, biomarker, blood pressure, hemodynamics, stenosis, valve

## Abstract

**Background—:**

Transvalvular peak pressure drops are routinely assessed noninvasively by echocardiography using the Bernoulli principle. However, the Bernoulli principle relies on several approximations that may not be appropriate, including that the majority of the pressure drop is because of the spatial acceleration of the blood flow, and the ejection jet is a single streamline (single peak velocity value).

**Methods and Results—:**

We assessed the accuracy of the Bernoulli principle to estimate the peak pressure drop at the aortic valve using 3-dimensional cardiovascular magnetic resonance flow data in 32 subjects. Reference pressure drops were computed from the flow field, accounting for the principles of physics (ie, the Navier–Stokes equations). Analysis of the pressure components confirmed that the spatial acceleration of the blood jet through the valve is most significant (accounting for 99% of the total drop in stenotic subjects). However, the Bernoulli formulation demonstrated a consistent overestimation of the transvalvular pressure (average of 54%, range 5%–136%) resulting from the use of a single peak velocity value, which neglects the velocity distribution across the aortic valve plane. This assumption was a source of uncontrolled variability.

**Conclusions—:**

The application of the Bernoulli formulation results in a clinically significant overestimation of peak pressure drops because of approximation of blood flow as a single streamline. A corrected formulation that accounts for the cross-sectional profile of the blood flow is proposed and adapted to both cardiovascular magnetic resonance and echocardiographic data.

## Abstract 2

Supplemental Digital Content is available in the text.

In the presence of aortic stenosis (AS), obstruction of the aortic outflow tract results in increased work of the left ventricle (LV) and eventually leads to heart failure if symptomatic severe AS is left untreated.^1^ The transvalvular pressure drop (TPD), also referred to as gradient in clinical guidelines, is the recommended measure of severity that best correlates with clinical outcomes.^2,3^ Continuous wave Doppler echocardiography and invasive catheterization measurements are the 2 main methodologies to assess the TPD, and despite underlying discrepancies between the approaches,^4,5^ clinical guidelines recommend the use of both methodologies interchangeably.^2,3^ Doppler-based pressure drops are typically evaluated noninvasively using the simplified Bernoulli (SB) formulation,^6^ which requires the assessment of the maximum velocity to estimate the peak instantaneous pressure drop at the point of maximum constriction or the mean drop during ejection. Catheter-based methodology provides 2 recordings of pressure before and after the obstruction and, therefore, estimates not the peak but the net pressure drop, by either the peak-to-peak difference (because synchronous acquisitions are not common) or by the mean drop of systolic pressure.^4,5^

**See Editorial by Fraser and Claus**

**See Clinical Perspective**

Despite its widespread use, the Bernoulli principle provides an oversimplification of human hemodynamics. The complete behavior of flow hemodynamics is described by the Navier–Stokes equations: the pressure drop is the result of the temporal acceleration of blood velocity (unsteady pressure component), the spatial transport of momentum of the blood (advective pressure component), and the deceleration because of friction losses (viscous pressure component). The Bernoulli principle is a simplification of the Navier–Stokes equations that estimates pressure drops between 2 locations across a cardiovascular compartment by applying 2 significant assumptions. The first is that the entire pressure drop is because of advective acceleration/deceleration of the blood flow, neglecting the impact of the unsteady and viscous components.^6,7^ The second is that blood flow is considered as a single streamline—or a column of flow with uniform velocity distribution—therefore, ignoring the complex hemodynamics.^8^ In an extended version of the Bernoulli principle used in hydraulics, the nonuniform velocity spatial distribution is handled by multiplying the estimated pressure drop by a correction factor *α* when the full profile is available.^9^ Nevertheless, the strict requirement of a complete acquisition of the velocity profile to evaluate this factor in the vasculature has hampered its clinical applicability to date.

In consideration of these aspects, a more accurate description of the intravascular pressure fields is now feasible through recent advances in medical imaging^10^ and computational methods.^11,12^ Using a combination of comprehensive velocity fields available via 4-dimensional (3D+time) flow phase-contrast cardiovascular magnetic resonance (4D flow CMR), and the work–energy relative pressure (WERP) estimation method,^13^ a more robust and accurate computation of pressure drops can be achieved. This formulation uses an energy principle derived directly from the Navier–Stokes equations, with a reduced number of simplifications, and enables the separate evaluation of each component of the pressure drop,^14^ accounting for the full 3D nature of the blood flow.

The aim of this work was to use the WERP approach to evaluate the 2 fundamental assumptions in the Bernoulli calculation for the assessment of the TPD and determine its accuracy in vivo. Accounting for the proximal velocity (as in a modified Bernoulli formulation) will not be a question visited in this work.

## Methods

### Patient Data

Thirty-two subjects with a bicuspid aortic valve were selected for this study from subjects undergoing CMR scans for another research study.^15^ The study protocol was approved by the West Berkshire ethics committee, and all participants or their guardians gave written informed consent. Each subject underwent a CMR scan on a 3T system (Trio, Siemens, Erlangen, Germany) for 4D flow CMR assessment using a 32-channel cardiac coil. Flow-sensitive gradient-echo pulse sequence CMR data sets were acquired with prospective ECG gating during free breathing using a respiratory navigator. The image acquisition volume was in an oblique sagittal plane encompassing the whole thoracic aorta, with voxel size 1.9–2.0×1.5–1.7×2.0–2.2 mm^3^ and temporal resolution 40 ms. The velocity-encoding range was determined using the lowest nonaliasing velocity on scout measurements (≤4.5 m/s in the most stenotic subject).

Subjects were divided between those with no significant AS (group I [n=20], mean TPD <20 mm Hg) and those with AS (group II [n=12], mean TPD >20 mm Hg) following current clinical guidelines.^2^ The Bernoulli method using the mean drop across the valve during systole was used for the computation of these pressure values. Aortic dimensions and hemodynamics data are shown in Table 1.

**Table 1.**
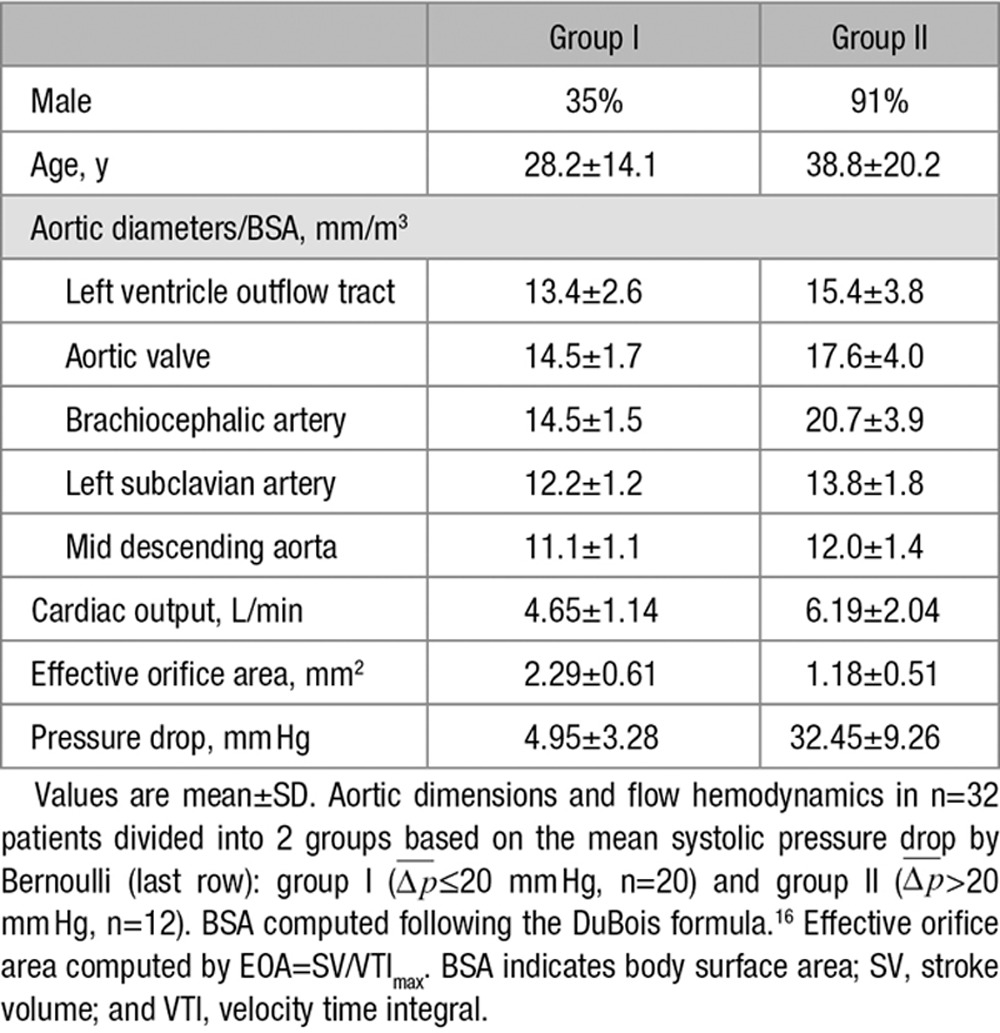
Aortic Dimensions and Hemodynamics

### Preprocessing and Definition of Anatomic Regions

4D flow CMR images had field inhomogeneities and eddy currents corrected using available preprocessing tools.^17^ The lumen of LV and aorta were identified, using a thresholding criterion calibrated by the peak velocity magnitude, to remove the impact of noise at the near-wall vascular regions. A skeletonization algorithm is then used to extract the centerline of the aorta and its perpendicular planes, as required for the WERP computations.

**Figure 1.**
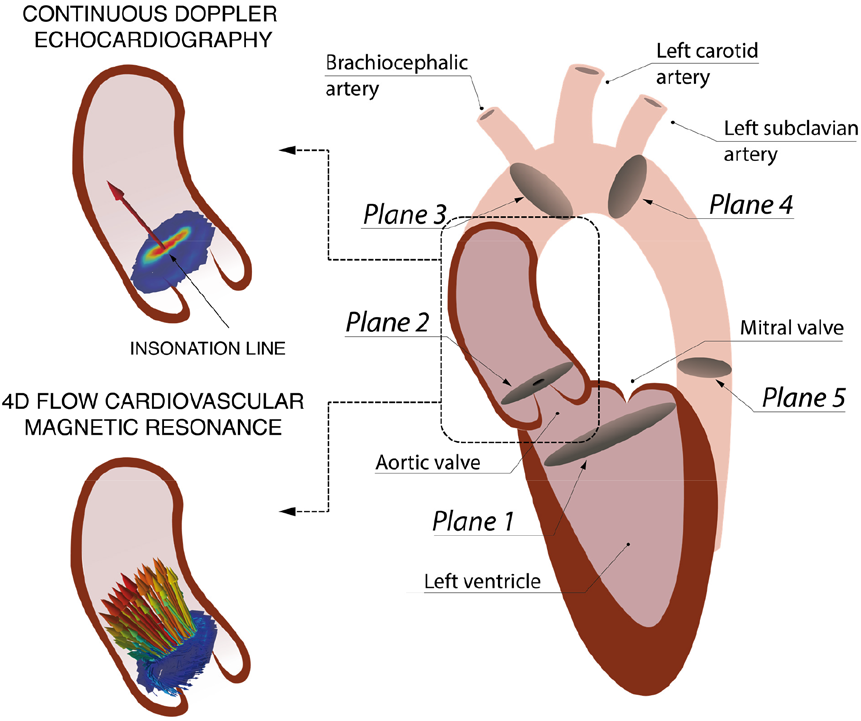
**Left**, Schematics of the velocity field at the vena contracta (VC) acquired during systole with continuous Doppler (1D encoded velocity value, **top**) and 4D flow cardiovascular magnetic resonance (CMR; 3D-encoded 2-dimensional velocity field, **bottom**). **Right**, Definition of the anatomic regions to compute the TPD from the left ventricular outflow tract (LVOT; plane 1) to the VC (plane 2). Two other anatomic regions are defined for the Material B in the Data Supplement, the ascending aorta (AA) from the VC to the brachiocephalic artery (plane 3) and the descending aorta (DA) from the left subclavian artery (plane 4) to a plane at the same height of the aortic valve plane (plane 5).

TPD were calculated over the transvalvular region (TVR), between the LV outflow tract (LVOT) (plane 1; Figure 1), and the vena contracta (VC; plane 2). The LVOT plane was located 12 mm before the VC, following the definition used by Garcia et al,^18^ and the VC is detected from the image as the plane containing the peak velocity magnitude, that is, the plane of maximum narrowing of the aortic valve jet.

### Simulated Doppler Echocardiography

To avoid intermodality variability in the interpretation of results, simulated echocardiographic velocity data were derived by sampling the 4D flow CMR data. Idealized conditions were taken: a perfect alignment between the direction of the blood jet and the ultrasound probe orientation, and no acoustic shadowing. Simulated echocardiographic data were then simply the peak velocity value in plane 2 at the VC (Figure 1), which was constructed through linear interpolation of the original 3D velocity field onto a grid of 1 mm×1 mm sample points in the perpendicular plane to the centerline of the aorta.

### Noninvasive Pressure Drop Estimates

The SB formulation^6^ only accounts for the advective pressure drop, assumes that the flow jet is a single streamline, and neglects the proximal velocity at the LVOT, approximating the pressure drop in mm Hg as


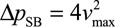
(1)

where *v*_max_ is the peak velocity at the VC, and the factor 4 comes from the conversion of pressure units from Pascals to mm Hg, taking a blood density of *ρ*=1060 kg/m^3^.

SB formulation neglects the unsteady and viscous terms of the Navier–Stokes equation; thus, we evaluated the magnitude of all the components of the pressure drop to determine if the assumption holds true. We used the WERP method, because of its accuracy and robustness,^13^ that computes the total pressure drop accounting for the complete fluid dynamics, that is, the unsteady, advective, and viscous components:


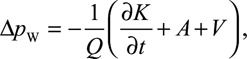
(2)

where *Q* is the flow rate computed at the outlet, 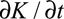 is the temporal derivative of the kinetic energy within the vascular region, *A* is the advective energy rate describing the energy transfer because of the physical movement of a fluid in and out of the domain, and *V* is the rate of viscous dissipation describing energy losses because of friction.

**Figure 2.**
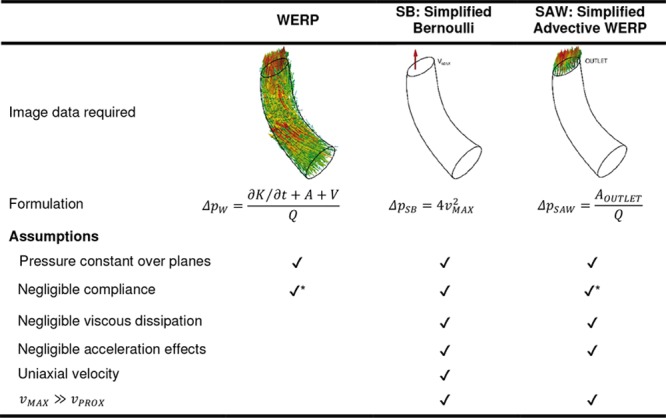
Mathematical formulations to compute a pressure drop. Compliant models can be added in the formulations labeled with (*), but this is not applied in this work. SAW indicates simplified advective WERP; SB, simplified Bernoulli; and WERP, work–energy relative pressure.

The assumption of spatially uniform velocity distribution was evaluated by a comparison of the pressure drop computed by SB to one accounting for the complete velocity profile at the VC, the simplified advective WERP (SAW) pressure drop (Δ*p*_SAW_)—see Material A in the Data Supplement for the derivation of SAW. SB, SAW, and WERP methods are schematically presented in Figure 2.

Within this work, we focus on instantaneous peak pressure drops at the VC and not on the net pressure drop downstream of the constriction. Results also include the temporal mean of this drop 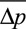 that is estimated averaging the 8 or 9 systolic frames of each subject.

### Statistical Analysis

Differences between groups I and II are evaluated by an unpaired *t* test.

## Results

### Analysis of the Components of the Pressure Drop

**Figure 3.**
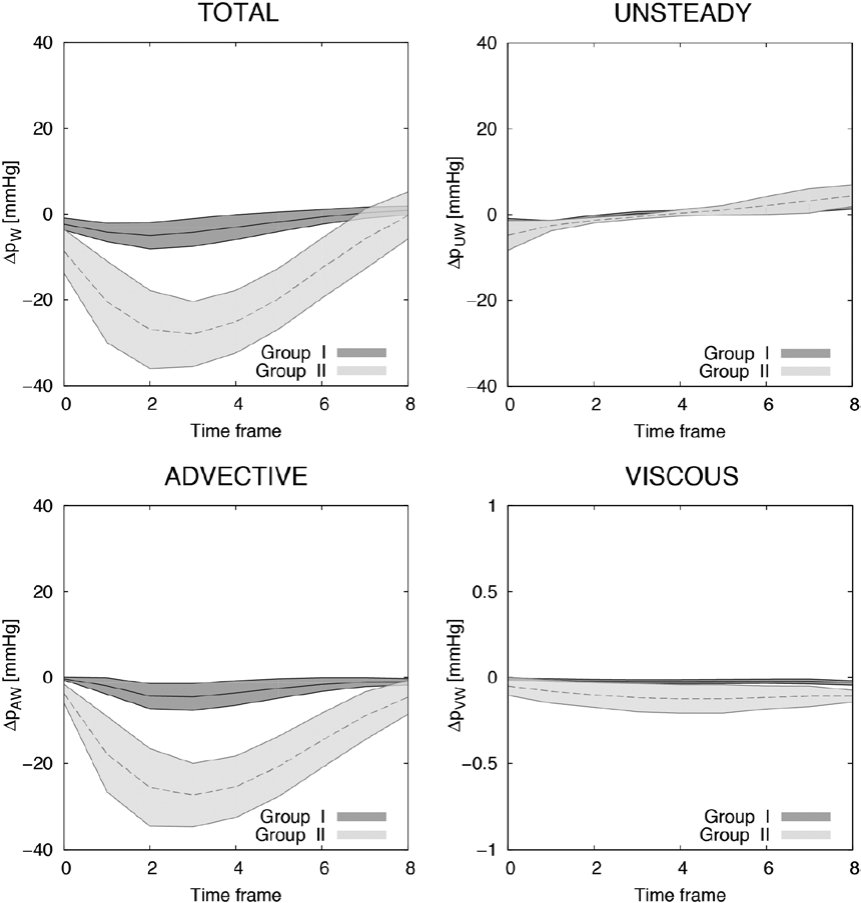
Instantaneous transvalvular pressure drop (TPD) and its components computed for group I (n=20) and group II (n=12) using work–energy relative pressure (WERP) formulation. Each line with range illustrates the mean±SD of the distribution.

The advective pressure component is the main contributor to the TPD, especially in higher degrees of stenosis (group II), as illustrated in Figure 3 and quantified in Table 2. Subjects in group II had a mean advective drop of 16.33±4.02 mm Hg, which reflected 99% of the mean total TPD on average (range 96%–101%) and was dominant over the unsteady component by almost 1 order of magnitude (2.09±1.44 mm Hg during acceleration) and over the viscous component by 2 orders of magnitude (0.10±0.06 mm Hg). Prevalence of the advective component is also shown in group I, although to a lesser extent (2.55±1.80, 1.49±0.57, and 0.02±0.01 mm Hg for the advective, unsteady, and viscous components, respectively).

**Table 2.**
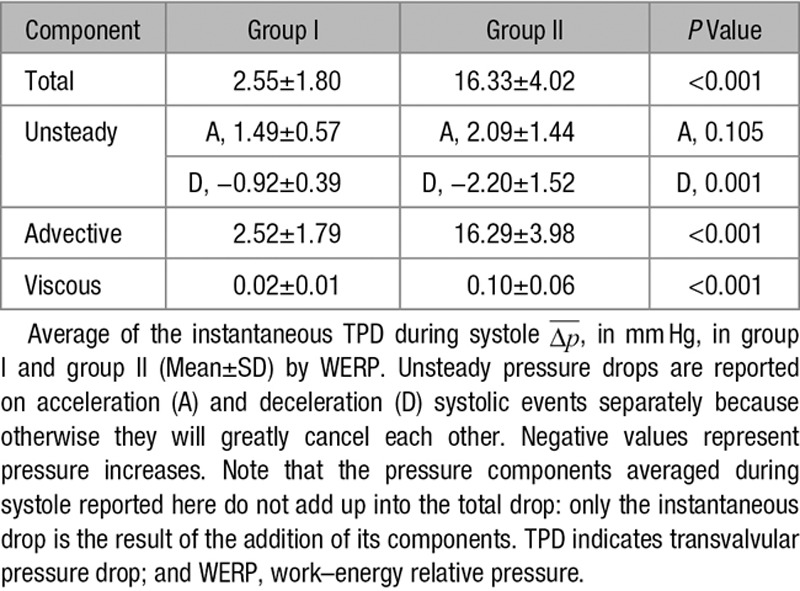
Pressure Drops and Components

Results in Table 2 highlight a clear differentiation in the TVR between groups for the advective and viscous drops (*P*<0.001) and for the unsteady component during deceleration (*P*=0.001), while showing nonsignificant differences during acceleration (*P*=0.105). To contextualize these results, Material B in the Data Supplement provides the pressure drops along the ascending and descending part of the aorta in the 2 experimental groups.

### Analysis of the Impact of the Velocity Profile in the Pressure Drop

The impact of the assumption of a flat velocity profile is assessed by comparing TPD computed using SB and SAW formulations, finding an SB overestimation of 54% in the 32 subjects (range 5%–136%), being smaller in the nonstenotic group (41% versus 76% for groups I and II, respectively).

**Figure 4.**
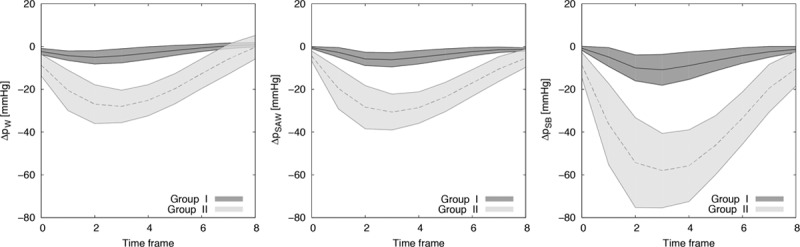
Instantaneous transvalvular pressure drop (TPD; mean±SD values during systolic frames) estimated for groups I and II using work–energy relative pressure (WERP; **left**), simplified advective WERP (SAW; **center**), and simplified Bernoulli (SB; **right**) formulations.

**Figure 5.**
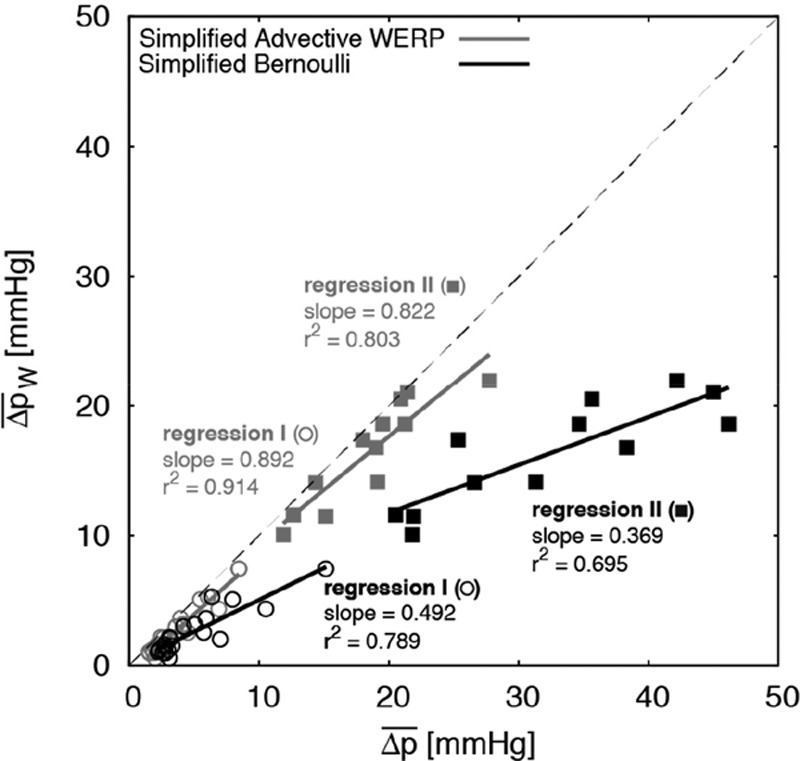
Linear regression between the reference mean transvalvular pressure drop (TPD) from 4D flow cardiovascular magnetic resonance (CMR) data using the work–energy relative pressure (WERP) formulation against the mean TPD estimated using the simplified Bernoulli (SB; black) and simplified advective WERP (SAW; gray) formulations in the 2 groups of patients. Case-specific values for subjects in group I (circles) and group II (squares), regressions for the estimation methods (solid lines), and identity line (dashed gray line).

Accounting for all the assumptions, Figure 4 illustrates the SB overestimation compared with the reference by WERP (average of 99% in stenotic subjects, range 49%–145%). SAW had a milder overestimation, averaging 14% (range 1%–35%) in the same group. Figure 5 reveals a poorer agreement with the reference pressure drops for SB when compared with SAW. SB also shows a lower precision (larger variability of the error) compared with SAW after correction for the linear regression observed in the 32 cases reported in Figure 5, with standard deviations observed for the 2 formulations of 0.8 and 0.5 mm Hg in group I and of 2.4 and 0.9 mm Hg in group II, respectively.

**Figure 6.**
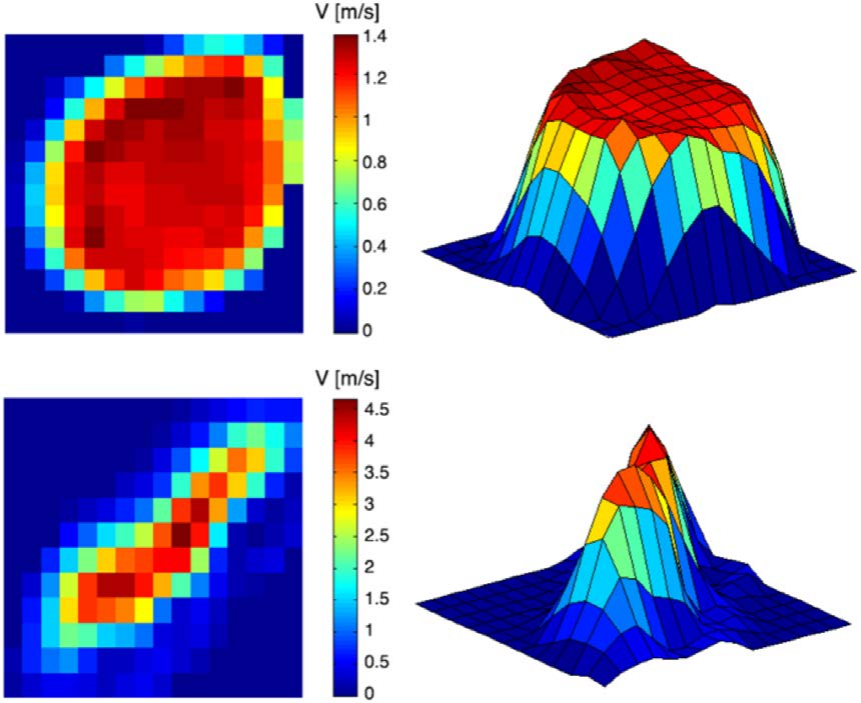
Velocity magnitude distribution from 4D flow data: in plane visualization (**left**) and 3D surface plot (**right**) in representative control (**top**) and stenotic (**bottom**) patients. The deviation from a flat profile in these 2 examples causes a simplified Bernoulli (SB) overestimation of a 20% and 136%, respectively.

To contextualize the impact of the velocity profile on the estimated pressure drops, 2 representative cases for patients in both groups are presented in Figure 6. For completeness, Material C in the Data Supplement provides a comprehensive description of the velocity profiles at the VC in all 32 subjects, demonstrating their wide variability. Material D in the Data Supplement provides an analysis of velocity profiles in 3 idealized stenoses, demonstrating that the SB overestimation as compared with SAW is uniquely caused by the velocity profile and illustrates that a paraboloid distribution introduces an overestimation of the advective drop of ≈100% (ie, double) by SB.

## Discussion

The noninvasive assessment of the peak TPD at the VC can be simplified to the computation of its advective component, consistent with the SB formulation. Nevertheless, our results report that this formulation introduces a variable overestimation (range of 5%–136% in 32 subjects) because velocity profiles at the VC are not uniform.

### Analysis of Pressure Components

We experimentally verified in vivo that the TPD is primarily driven by the spatial acceleration of the flow. This confirms the sensible choice of the SB formulation to quantify the maximal pressure drop from continuous Doppler recordings because the Bernoulli principle simplifies the flow through a pipe by only accounting for the advective forces. This result agrees with the seminal work by Hatle et al^6^ that established a landmark piece of evidence to justify the adoption of the SB formulation to stratify vascular constrictions.

However, the simplification of the pressure drop into only the advective component is not generalizable to all anatomic regions. We demonstrated that in the descending aorta—without any obstruction—the pressure drop is dominated by the unsteady component (see Material B in the Data Supplement), in agreement with results reported in the human healthy aorta.^14^ The transmitral pressure drop has been shown, contradicting the initial evidence,^6^ to require the unsteady component to complement the SB formulation to find a good agreement with catheterization recordings.^19^ The unsteady component also plays a significant role in the TPD in the pulmonary valve, and neglecting it with the SB formulation leads to a significant underestimation of the pressure drop.^20^

The ability to analyze the contributors of a pressure drop also opens the possibility for an improved understanding of the impact of the valve dysfunction and to eventually define biomarkers with enhanced risk stratification and predictive power. Bernoulli-based metrics from clinical guidelines^2,3^ only capture the advective drop in the TVR, and our analysis reveals the presence of additional contributors to the functional differences between a stenotic and healthy valve. First, a stenotic valve introduces a significant increment of the laminar viscous losses in all vascular segments analyzed (Table 2; Material B in the Data Supplement). Viscous drops capture the inefficiency of the aorta as a conduit, an additional burden to the heart in every heartbeat and, therefore, could be a more specific prognostic marker for heart failure. It is, nevertheless, important to highlight that current spatial resolution of phase-contrast CMR provides an underestimated and resolution-dependent viscous dissipation, up to only a 9% of the real magnitude with isotropic resolution of 2 mm.^21^ We speculate that by using similar CMR protocols across studies, viscous dissipation can be estimated with sufficient precision to enable the extraction of a clinically diagnostic value. The differences found in this study, together with previous findings of the analysis of viscous laminar losses,^22^ support this claim.

Results also reveal that the narrow jet produced by a stenotic valve introduces a significantly larger unsteady pressure drop in the ascending aorta (AA; Material B in the Data Supplement). The heart requires more energy to create the flow momentum of the narrow blood jet caused by a stenotic valve and to accelerate it in time, and this functional difference might be a specific prognostic marker for heart failure. It is relevant to note that this increment in unsteady pressure cannot be captured by the peak or the average pressure drop value: an average during systolic events will cancel the acceleration and deceleration events, and the peak unsteady effects are not synchronous with the peak advective effects^14^ and, therefore, not contributing to the peak TPD value in the stenotic group that is dominated by advective effects. Further studies are, thus, required to identify which pressure component holds the largest prognostic value.

### Impact of the Velocity Profile

The most interesting finding of this study is that the SB simplification of blood flow as a single streamline^8,23^ produces a significant overestimation of the estimated pressure drop. The study in Material D in the Data Supplement demonstrates that SB would only be accurate if the velocity distribution was uniform, with all particles at the cross-section of a vessel having the same velocity.

Analysis of our 32 subjects reveals a large variability in the morphology of the velocity profiles, as illustrated in Material C in the Data Supplement. The nonstenotic group shows flatter velocity profiles and had a reduced overestimation by SB as compared with the stenotic group. This finding agrees with previous works that already describe the overamplification of the assumption of a nonuniform velocity profile^24,25^ and attributed the variability of the measurements to the different flow profile characteristics from patient to patient.

The cause of this variability could be initially attributed to the shape of the valve orifice: the more circular shape, the blunter the velocity profile. This was the justification in the early studies that tested and verified the validity of Bernoulli principle, despite irregular orifice shapes tested.^26,27^ Nevertheless, it is the blood velocity distribution, and not the shape of the orifice, that should be analyzed. In these preliminary works, the abrupt transition from a wide cavity into a small orifice is not fully representative of the cardiac valve mechanics. We speculate that the interaction between a pulsatile flow and the deformable and compliant valve leaflets that create the gradual transition from the ventricular chamber to the blood jet is the main cause of the nonflat velocity profiles in valve stenosis.

The core of the question then is to interrogate the velocity profile at the point of the VC: any deviation from a flat shape is a cause of overestimation of SB. And the existence of nonflat velocity profiles at the VC has been reported using a variety of technologies, such as advanced laser particle tracking technologies,^28^ Doppler ultrasound,^29,30^ and phase-contrast CMR.^31^ Nevertheless, it should be noted that the point-spread function of a CMR system causes a spatial averaging of the velocity data. As a consequence, our results contain a spurious source of amplification of the deviation from a flat velocity profile. This factor alone cannot explain the anisotropic velocity distribution highlighted at the VC of the representative stenotic case illustrated in Figure 6, nor the wide range of shapes in the velocity profiles observed in Material C in the Data Supplement. Previous experimental findings in bioprosthetic valves comparing peak drops at the VC between manometers and Bernoulli-based continuous Doppler assessment reported an average overestimation of 24% with the latter (average slope of 0.809 in all explanted valves in Table 5 in Stewart et al^32^), which is approximately half of the 54% in our findings. Future work is, thus, needed to fully characterize the overestimation of the advective pressure drop at the VC through the acquisition of more accurate velocity profiles.

The simplification of the transvalvular jet as a flow field with uniform velocity distribution, therefore, introduces a loss not only in accuracy but also in precision. Average bias correction of SB (our results suggest a factor of 0.65 to compensate the average 54% of overestimation) will not be enough to account for the fact that the increment of work, or energy, to push blood through the valve does depend on the morphology of the blood jet.

### Potential Correction of the Bernoulli Method

The proposed method (SAW formulation), by correctly accounting for the factor of the velocity profile, can improve the risk stratification of any condition that currently relies on Bernoulli’s simplification. AS is the condition exemplified and analyzed in this work, and an immediate extension is the functional characterization of the narrow LVOT in hypertrophic cardiomyopathy.

The SAW formulation (see details in Material A in the Data Supplement) is conceptually an extension of SB into a cross-section of the vessel. As such, it can also be extended to account for the proximal velocity, as detailed in Material A in the Data Supplement. SAW can be used with both 4D and 2D flow CMR because data are only required in one plane, thus, enabling the possibility of high frame rates of 2D acquisitions. The adoption of the correct formulation is, therefore, straightforward in future clinical research studies using CMR flow.

SAW introduces further requirements in the spatiotemporal resolution of velocity data compared with SB. It has been reported that the relative error of the total pressure drop was below 12% with a coarse temporal resolution of 8 frames per heartbeat (125 ms of temporal resolution) and with a reasonable 2 mm of spatial resolution and 20 dB of signal to noise ratio.^13^ Note that the technique used in this work has higher frame rates (40 ms of temporal resolution). Further research is, nevertheless, needed to identify the optimal CMR acquisition protocol (resolution and noise) for SAW.

Access to 4D flow CMR sequences is mainly restricted to specialized research centers, and translation of our findings to echocardiographic imaging is strongly desirable. A CMR technique may help to develop an echo protocol to address this area better, potentially with newer techniques in 3D echo flow, based on the positive feasibility results reported in this article. In this direction, Material A in the Data Supplement describes how to adapt SAW to the characteristics of the velocity data obtained by echocardiography. Furthermore, Material E in the Data Supplement illustrates that simulated 3D echocardiographic data, offering a complete velocity profile at the VC with artifacts from the funneling effect and from the projection of the velocity along the echocardiographic probe insonation line, will introduce a tolerable bias. Access to one line of insonation as with a 2D echo probe will only partially correct SB overestimation and will suffer from an additional variability caused by a partial view of the complete profile, justifying the need of improved acquisition strategies that render a more complete picture of the velocity profile. Besides these theoretical considerations, practical considerations, such as the limited access to the valve anatomy by shadowing effects caused by a calcified valve, or the presence of aliasing, will be additional challenges that need to be addressed for the successful adoption of a correct estimation of the advective pressure drop at the VC.

The adoption of the improved formulation is, thus, feasible to a wide range of imaging acquisition protocols and modalities, and further research is needed to define the optimal strategy to control the location of the VC to be imaged, to identify the direction of the jet, and to maximize the amount and quality of velocity data.

### Peak Versus Net Pressure Drops

Our work focused on the analysis of the peak pressure drop at the point of the VC and not on the net pressure drop after the VC, which has been proposed as a more efficient biomarker for the degree of constriction experienced by the blood flow, that is, the additional burden that the LV has to overcome.^27,33,34^ The net pressure drop is lower than the peak pressure drop estimated at the TVR, and it better correlates with catheter measurements.^4,5^ It accounts for the partial recovery of pressure downstream of the VC^18,33,34^ caused by the full recovery of the advective pressure (ie, the transition from a narrow jet to a wide velocity profile across the complete aortic cross-section) and by the losses because of viscous dissipation.

The net drop can be estimated noninvasively from the peak drop (peak velocity) and an assessment of the amount of the energy loss as a function of the valve effective orifice area and the size of the AA.^33^ Here, we speculate that a more accurate and robust estimation of the peak drop, as demonstrated in this work, will also improve the prediction of the net pressure drop from velocity and geometric data.

The net drop quantifies pressure differences between LVOT and end of the AA, and our results provide further insights about the choice of the anatomic point after the VC where to estimate the net drop. In our cohort, the advective pressure drop in the TVR was fully recovered along the AA in both groups (Material B in the Data Supplement). The length of the AA may, thus, be enough to make the transition from a narrow jet of the VC to a fully developed flow profile, but further investigation in more severe stenotic subjects is needed to confirm this finding. On the contrary, results report that the length of the vascular domain that is affected by additional viscous losses caused by the constriction is larger than the AA: losses in the DA are doubled in the stenotic group compared with the control group (0.15 versus 0.07 mm Hg; *P*<0.001; Material B in the Data Supplement), accounting for approximately a quarter of the cumulative viscous pressure drop in the 3 regions under study. Quantification of the total additional burden caused to the heart by a stenotic valve might, thus, require the study of the complete aortic anatomy, and not only the AA.

### Limitations

The main limitation is the lack of catheterization recordings of pressure and is justified by the experimental difficulty to get the instantaneous pressure drop between the LVOT and the VC in vivo. This requires a stable and accurate placement of the catheterized sensor at the VC to avoid the spurious effect of the pressure recovery and the verification that the sensor is not introducing an artifact in the pressure data, as it is expected in the narrow jets.^35^

Current spatiotemporal resolution of phase-contrast CMR data is not suitable for the estimation of the net pressure drop because it misses the energy loss caused by laminar viscous or turbulent dissipation.^21,36^ An attenuated and resolution-dependent version of the real dissipation because of laminar friction effects and a surrogate of the turbulent viscous dissipation are the metrics that can be extracted from this data.^21,36^

## Sources of Funding

The study was supported by Wellcome Trust and Royal Society (Grant no. 099973/Z/12/Z, WT088641), EPSRC (EP/N011554/1), British Heart Foundation Centres of Research Excelence (KCL and Oxford), and UK NIHR (Guy’s and St Thomas’ & Oxford NIHR Biomedical Research Centres, Healthcare Technology Co-operative for Cardiovascular Disease).

## Disclosures

None.

## Supplementary Material

## CLINICAL PERSPECTIVE

A more accurate and precise noninvasive estimation of the peak pressure drop, beyond Bernoulli’s principle, is now possible by a more comprehensive examination of blood velocity. This work illustrates the large variability in the shape of the velocity profile at the vena contracta after a stenosed valve and how this introduces an uncontrolled source of error in current practice based on Bernoulli’s formulation. Errors are larger and more variable in stenotic cases, where the narrow and irregular opening of the impaired valve produces anisotropic, nonaxisymmetric, and skewed blood flow jets. Controlling this factor through the formulation proposed in this work will improve risk stratification and clinical decision-making in valve stenosis and potentially in any other conditions that experience flow constriction, such as a narrowed left ventricular outflow tract in hypertrophic cardiomyopathy. More precise and accurate values of pressure drop can already be obtained clinically from cardiovascular magnetic resonance, also removing the difficulty of aligning an echo probe with the flow jet. Widespread clinical adoption using echo probes is feasible, direct for 3D echo with a good anatomic window, although further efforts to define the optimal acquisition strategy are needed for 2D echo. Avenues of further research are now open to establish the new cutoff values of pressure drop to risk-stratify flow constrictions, to improve the prediction of the net pressure drop, and to determine which pressure biomarker (peak, net, or any of its components) holds the largest prognostic value to predict clinical outcomes.
