# Supplementary material for: Beyond Bernoulli: Improving the Accuracy and Precision of Noninvasive Estimation of Peak Pressure Drops
Source: Circ Cardiovasc Imaging. 2017 Jan 16;10(1):e005207. doi: 10.1161/CIRCIMAGING.116.005207 (PMC5265685; doi:10.1161/CIRCIMAGING.116.005207)
Supplement: Supplementary file 1 [file hci-10-e005207-s001.pdf]

Supplemental Material

A. Mathematical details of the WERP method

The original form of the WERP formulation is defined from the Navier-Stokes equation based on the work-energy principle, see Donati *et al.*[1], and estimates the pressure drop Δp_W across the vascular region Ω with inlet plane Γ_{INLET} and outlet plane Γ_{OUTLET} defined from the lumen segmented from the flow data as,

$$\Delta p_W = \frac{1}{Q}\left(\frac{\partial K}{\partial t} + A + V\right). \qquad \text{(Equation A.1)}$$

The blood flow rate Q, the kinetic energy K, the advective energy rate A and the viscous dissipation rate V can be evaluated by solving numerical surface and volume integrals as,

$$\begin{aligned}
Q &= \int_{\Gamma_{OUTLET}} \boldsymbol{v}\cdot\boldsymbol{n}\,d\boldsymbol{x} = -\int_{\Gamma_{INLET}} \boldsymbol{v}\cdot\boldsymbol{n}\,d\boldsymbol{x}, && [m^3/s]\\
K &= \frac{\rho}{2}\int_{\Omega} \boldsymbol{v}\cdot\boldsymbol{v}\,d\boldsymbol{x}, && [Pa\,m^3]\\
A &= \frac{\rho}{2}\left(\int_{\Gamma_{INLET}} |\boldsymbol{v}|^2(\boldsymbol{v}\cdot\boldsymbol{n})\,d\boldsymbol{x} + \int_{\Gamma_{OUTLET}} |\boldsymbol{v}|^2(\boldsymbol{v}\cdot\boldsymbol{n})\,d\boldsymbol{x}\right), && [Pa\,m^3/s]\\
V &= \frac{\mu}{2}\int_{\Omega} D(\boldsymbol{v}):D(\boldsymbol{v})\,d\boldsymbol{x}, && [Pa\,m^3/s]
\end{aligned} \qquad \text{(Equation A.2)}$$

where $\boldsymbol{v}$ is the three-dimensional time-dependent velocity field at the generic voxel, $\boldsymbol{n}$ is the normal direction on the inlet/outlet plane, $\rho = 1060\ \mathrm{kg/m^3}$ and $\mu = 0.004\ \mathrm{Pa\cdot s}$ are the blood density and dynamic viscosity, respectively, and $D(\cdot) = [\nabla(\cdot) + \nabla(\cdot)^T]$. Note that $d\boldsymbol{x}$ refers to the variable of integration, i.e. $d\boldsymbol{x} = d\mathcal{V}$ for volume integrals (kinetic and viscous contributions computed over volume Ω) and $d\boldsymbol{x} = d\mathcal{S}$ for surface integrals (flow and advective contributions computed over surface Γ). Using separation of the pressure components the complete advective pressure drop evaluated using the WERP method $\Delta p_{AW} = A/Q$ yields from Equation A.2,

$$\Delta p_{AW} = \frac{\rho}{2}\left(\int_{\Gamma_{INLET}} |\boldsymbol{v}|^2(\boldsymbol{v}\cdot\boldsymbol{n})\,d\boldsymbol{x} + \int_{\Gamma_{OUTLET}} |\boldsymbol{v}|^2(\boldsymbol{v}\cdot\boldsymbol{n})\,d\boldsymbol{x}\right)\Big/\int_{\Gamma_{OUTLET}} \boldsymbol{v}\cdot\boldsymbol{n}\,d\boldsymbol{x}, \qquad \text{(Equation A.3)}$$

therefore reducing the drop computation to surface integrals on the inlet and outlet planes and making it applicable to 2D CMR or 3D Doppler echocardiographic data.

Equation A.3 can be further simplified by assuming outlet velocities much larger than inlet velocities (which is likely to hold in the transvalvular region defined from the LVOT to the VC, especially in stenosed cases), defining the SAW (Simplified Advective WERP) formulation:

$$\Delta p_{SAW} = \frac{\rho}{2}\left(\int_{\Gamma_{OUTLET}} |\boldsymbol{v}|^2(\boldsymbol{v}\cdot\boldsymbol{n})\,d\boldsymbol{x}\right)\Big/\int_{\Gamma_{OUTLET}} \boldsymbol{v}\cdot\boldsymbol{n}\,d\boldsymbol{x}, \qquad \text{(Equation A.4)}$$

The SAW approach can be further reduced to estimate the advective energy rate from velocity values along of the VC along a single line, not in the complete perpendicular plane, thus enabling applicability to 2D echocardiographic images. Equation A.4, by replacing the surface integrals at the outlet plane for line integrals along the line λ defined by intersecting the hypothetical insonation plane with the outlet plane of the aortic lumen plane, and by considering the fact that velocity values are already projected in the direction of the line of insonation, can be rearranged as,

$$\Delta p_{SAW} = \frac{\rho}{2}\left(\int_{\lambda} (|\boldsymbol{v}|^2 \cdot \boldsymbol{v})\, d\boldsymbol{x}\right) \Big/ \int_{\lambda} \boldsymbol{v}\, d\boldsymbol{x}, \qquad \text{(Equation A.5)}$$

It is worth noting that advective WERP and Bernoulli formulations are similar - as they both characterize the pressure drop using advective effects - and the mathematical link between them is here explained. In the WERP approach, the blood flow rate Q can be indifferently estimated at the inlet or outlet planes defined from the image data as,

$$Q = \int_{\Gamma} \boldsymbol{v} \cdot \boldsymbol{n}\, d\boldsymbol{x} = v_{MAX}\Psi \qquad \text{(Equation A.6)}$$

Here, v_{MAX} is the maximum velocity at the inlet/outlet plane and $\Psi = \int_{\Gamma} \Phi\, d\boldsymbol{x}$, where Φ is the normalized shape function in the normal direction for the inlet/outlet velocity profile. By substituting the advective WERP formulation in Equation A.3 into Equation A.6, the following yields,

$$-\Delta p_{AW} Q + \frac{\rho}{2}\left(\int_{\Gamma_{INLET}} v_{MAX,INLET}^3\, d\boldsymbol{x} + \int_{\Gamma_{OUTLET}} v_{MAX,OUTLET}^3\, d\boldsymbol{x}\right) = 0. \qquad \text{(Equation A.7)}$$

If we assume velocity at the planes aligned to the planes normal $\boldsymbol{n}$, substitution of Equation A.6 (selectively evaluated at the inlet/outlet planes) into Equation A.7 yields,

$$\Delta p_{AW} = \frac{\rho}{2}\left(v_{MAX,OUTLET}^2 \mathrm{Z}_{OUTLET} - v_{MAX,INLET}^2 \mathrm{Z}_{INLET}\right), \qquad \text{(Equation A.8)}$$

where $\mathrm{Z}_{INLET} = \int_{\Gamma_{INLET}} \Phi_{INLET}^3\, d\boldsymbol{x}/\Psi_{INLET}$ and $\mathrm{Z}_{OUTLET} = \int_{\Gamma_{OUTLET}} \Phi_{OUTLET}^3\, d\boldsymbol{x}/\Psi_{OUTLET}$ are functions depending on the normalized profile shape only. Consequently, in the hypothesis of a flat velocity profile (i.e. $v_{OUTLET} = v_{MAX,OUTLET}$ and $v_{INLET} = v_{MAX,INLET}$ as in Bernoulli based formulations) and with blood density $\rho = 1060\ \mathrm{kg/m^3}$, Equation A.8 simplifies to the corrected Bernoulli formulation $\Delta p_{CB} = 4(v_{OUTLET}^2 - v_{INLET}^2)$. Finally, the SAW pressure drop can be obtained from Equation A.8, by assuming that $v_{OUTLET} \gg v_{INLET}$, therefore yielding,

$$\Delta p_{SAW} = \frac{\rho}{2} v_{MAX,OUTLET}^2 \mathrm{Z}_{OUTLET}, \qquad \text{(Equation A.9)}$$

that assuming a flat velocity profile yields the SB pressure drop $\Delta p_{SB} = 4v_{OUTLET}^2$.

B. Pressure drop in AA and DA

To offer a comparative analysis of the contribution of pressure drop components in different vascular segments, computations are also performed in the Ascending Aorta (AA) - from the VC (Plane 2, see Figure 1 in main manuscript) to the brachiocephalic artery (Plane 3) - and the Descending Aorta (DA) - from the left subclavian artery (Plane 4) to a plane at the same height of the aortic valve (Plane 5).

A clear differentiation between groups is revealed in the AA for all pressure components (see Table A1 and Figure A1). Compared to the TVR, in the AA the advective component dominates over the viscous-driven drop by approximately two orders of magnitude in Group II, but the AA shows an increased impact of the unsteady term to the total drop. In the DA differences are still present, with stenotic subjects experiencing higher pressure losses due to viscous effects (0.15 vs 0.07 mmHg, $p<0.001$). In the DA, the total pressure drop experiences a sensible decrease in Group II caused by the absence of abrupt variations in the aortic geometry. In both groups the unsteady term becomes prevalent over the others.

Results also report how the widening of the aortic flow jet downstream of the VC captured in the AA causes a recovery of the advective TPD in subjects in Group II, with pressure drop magnitudes comparable with those observed in the TVR (-16.09 and 16.33 mmHg in AA and TVR, respectively), but with opposite sign.

AA			DA		
**Group I**	**Group II**	**p-value**	**Group I**	**Group II**	**p-value**
-2.02 ± 1.59	-15.94 ± 4.41	< 0.001	**A** 3.36 ± 0.82<br>**D** **-**3.26 ± 1.48	**A** 3.41 ± 0.92<br>**D** **-**3.18 ± 0.86	**A** 0.887<br>**D** 0.881
**A** 3.96 ± 2.70<br>**D** -2.37 ± 1.00	**A** 8.76 ± 3.82<br>**D** -7.02 ± 2.34	**A** < 0.001<br>**D** < 0.001	**A** 3.70 ± 0.93<br>**D** **-**3.51 ± 1.65	**A** 3.38 ± 0.90<br>**D** **-**3.37 ± 0.65	**A** 0.358<br>**D** 0.787
-2.14 ± 1.79	-16.09 ± 4.54	< 0.001	-0.09 ± 0.69	-0.58 ± 0.74	0.069
0.06 ± 0.03	0.33 ± 0.11	< 0.001	0.07 ± 0.03	0.15 ± 0.06	< 0.001

Table A1 - Average of the instantaneous pressure drop during systole $\overline{\Delta p}$, in mmHg, in Group I and Group II (Mean±std) in the ascending and descending aorta (AA and DA respectively). Differences evaluated by an unpaired T-test. Unsteady pressure drops are reported on acceleration (A) and deceleration (D) systolic events separately because otherwise they will greatly cancel each other. Negative values represent pressure increases. Note that the pressure components averaged during systole reported here do not add up into the total drop: only the instantaneous drop is the result of the addition of its components.

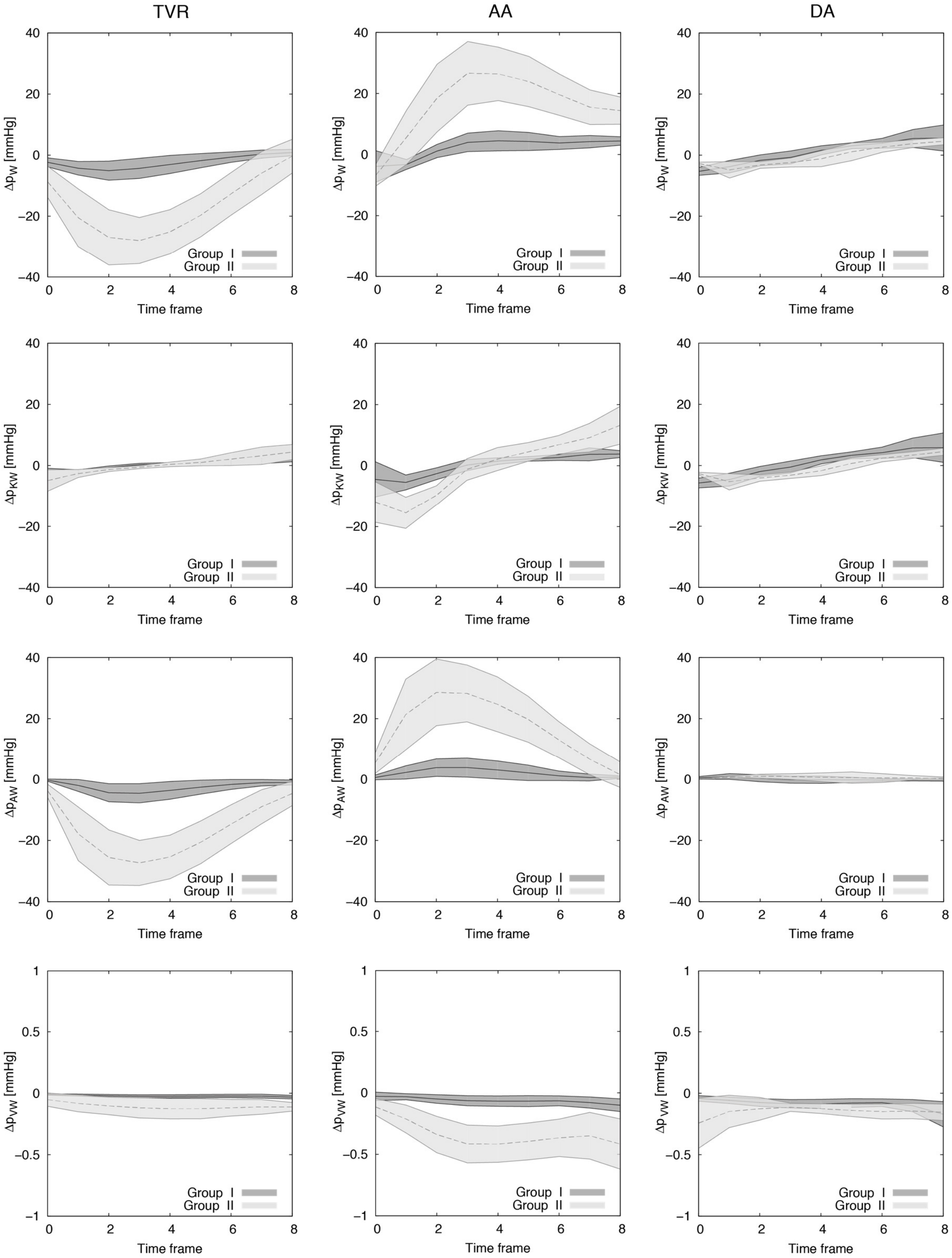

Figure A1 - Temporal transients of the instantaneous pressure drops computed for Group I (n=20, dark gray) and Group II (n=12, light gray) in the TVR (left), in the AA (center) and in the DA (right) using the WERP formulation. Each transient illustrates the mean±std of the distribution. From top to bottom: total, unsteady, advective and viscous pressure drops.

C. Velocity profiles at the vena contracta from 4D flow CMR

The velocity profiles from 4D flow CMR data at the VC are shown in Figures A2 and A3, with 2D surface plots of the velocity magnitude field and 12 different 1D velocity curves extracted for each case. The 2D velocity profiles are generally blunt, with peak velocities $v_{MAX} < 2.5\ \mathrm{m/s}$, for subjects in Group I, clearly showing a reduced variability of the 1D curves when compared to subjects in Group II, where the peak velocities are $v_{MAX} \geq 2.5\ \mathrm{m/s}$.

In addition, the departure from the uniform velocity distribution at the VC is quantified from the image data in terms of the kinetic energy correction factor α [2], estimated as,

$$\alpha = \frac{1}{\mathrm{A}} \int_{\mathrm{A}} \left(\frac{v(i,j)}{\bar{V}} \right)^3 d\mathrm{A}, \qquad \text{(Equation C.1)}$$

where $v(i,j)$ is the velocity value at pixel (i,j) and $\bar{V}$ is the average velocity across the VC of area A. Note that a uniform and a parabolic velocity distribution would imply $\alpha = 1$ and $\alpha = 2$, respectively. α is consistently higher in stenosed (2.03 ± 0.18) compared to control patients (1.52 ± 0.16).

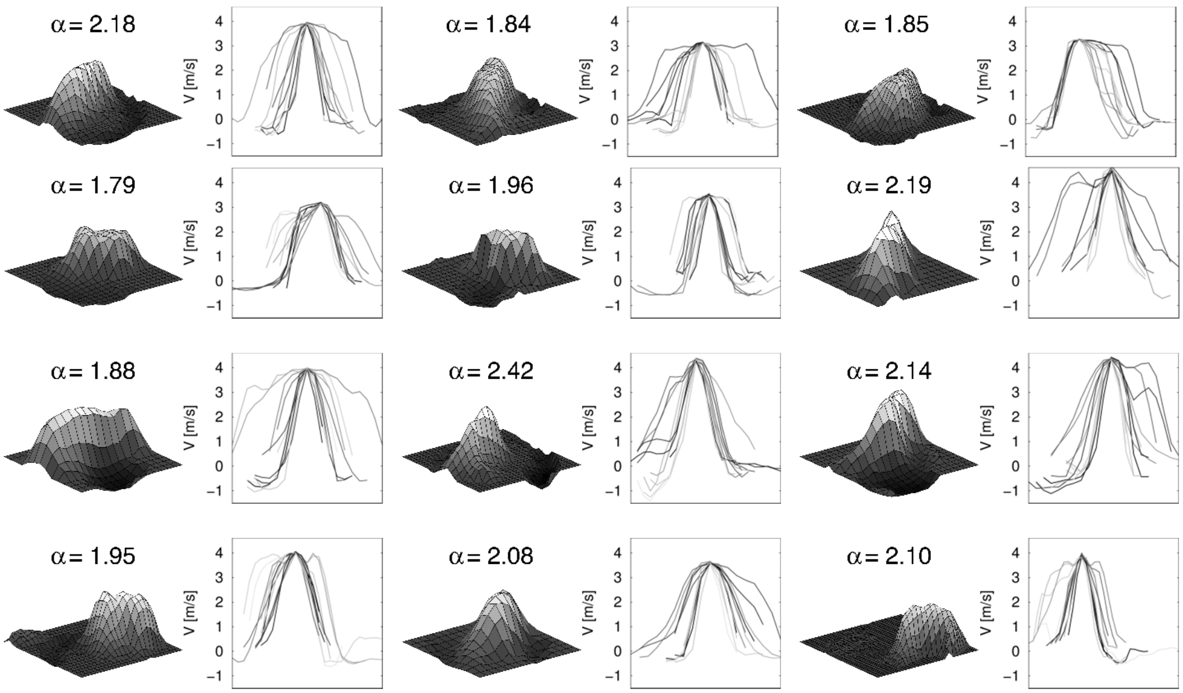

Figure A2 - Velocity profiles from 4D flow data of the 12 stenotic subjects (Group II) and correction factor α.

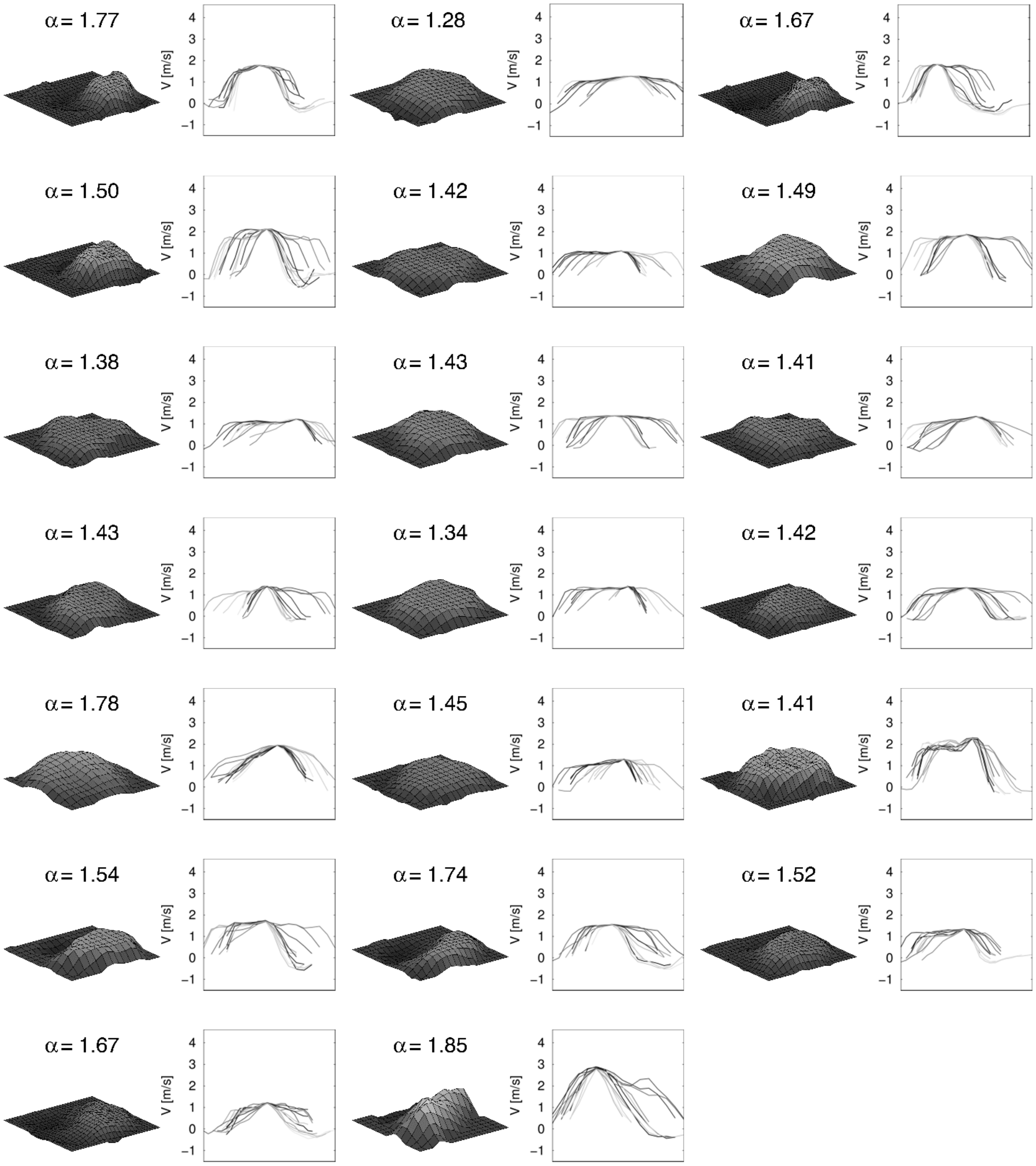

Figure A3 - Velocity profiles from 4D flow data of the 20 non-stenotic subjects (Group I) and correction factor α.

D. The impact of the velocity profile on the pressure drop estimation

The observed difference between SAW and Bernoulli formulations is experimentally verified with an *in silico* study. A steady flow on a straight tube with a change of diameter is simulated in a computer. Inlet and outlet velocity fields $v(x, y)$ are imposed analytically using the generic formula for poweroids,

$$v(x,y) = v_{MAX}\left(1 - \frac{((x - x_C)^2 + (y - y_C)^2)^{\beta/2}}{R^\beta}\right), \quad \text{(Equation D.1)}$$

where v_{MAX} is the peak velocity, x_C and y_C are the coordinates of the center, R is the radius and β is a coefficient accounting for the shape of the profile. We define a reference case, by choosing the pipe dimensions and flow properties such as the cardiac output $CO = 5\ \mathrm{L/m}$, the ratio between outlet and inlet radii $R_{OUTLET}/R_{INLET} = 0.25$, the density $\rho = 1060\ \mathrm{kg/m^3}$ and viscosity $\mu = 0.004\ \mathrm{Pa \cdot s}$ to be representative of those in the human thoracic aorta in the presence of AS. Additionally, we select a spatial resolution $dx = 0.5\ \mathrm{mm}$ and a velocity shape coefficient $\beta = 4$ to reproduce a quasi-paraboloidal profile. We thus compare the ratio of pressure drops estimated with the SB and SAW formulations, selectively testing: (1) the impact of the cardiac output ($CO = 4\ \mathrm{L/m}$ and $CO = 6\ \mathrm{L/m}$), (2) the stenosis level in terms of the ratio between radii ($R_{OUTLET}/R_{INLET} = 0.125$ and $R_{OUTLET}/R_{INLET} = 0.5$), (3) the spatial resolution ($dx = 0.25\ \mathrm{mm}$ and $dx = 1\ \mathrm{mm}$) and the (4) shape of the velocity profile in terms of the shape coefficient, in order to reproduce configurations that are likely to be found in the human aorta, spanning from paraboloidal ($\beta = 2$) to blunt profiles ($\beta = 10$), see Figure A4.

Results show a global overestimation obtained with the Bernoulli approach, independent of the spatial discretization, the outlet/inlet radii ratio or imposed flow rate. On the contrary, the difference between SAW and Bernoulli estimates is highly dependent on the shape of the 3D velocity profile, with the minimum gap obtained with blunt profiles.

Note that the three velocity profiles described in this in-silico workbench can be representative of 3 subjects. If these 3 subjects had the same peak velocity, they would have the same level of AS severity assessed by SB, but in reality the advective pressure drop could be quite different among them, and this difference would be noticeable accounting for the complete velocity profile (i.e. through a SAW formulation).

To better illustrate the impact of the velocity profile in the pressure drop estimation, we have selected two representative cases from our cohort in Figure A5. These two cases showed a large difference in AS severity as assessed by SB, when in reality, accounting for the complete velocity profile through SAW, the

two cases did have a similar pressure drop. This example illustrates how correctly accounting for the physics of the blood flow when computing the advective pressure drop removes a source of error caused by SB simplification.

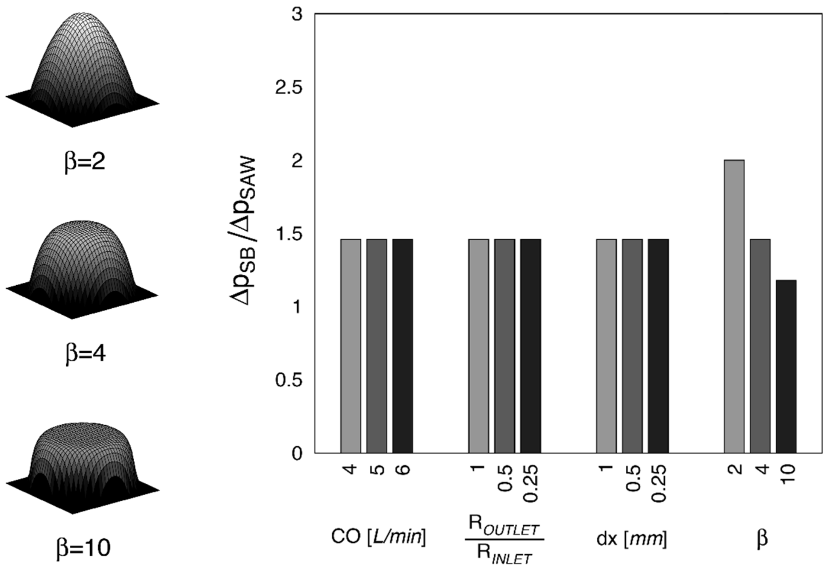

Figure A4 – Determinants of over-estimation by Bernoulli on a computational (in silico) workbench of a 3D straight pipe with steady velocity field. Left panel: representation of velocity profiles at the outlet (at the VC) with different velocity shape coefficients β. Right panel: pressure drop ratio between drops estimated with the simplified Bernoulli (SB) and simplified advective WERP (SAW) formulations ($\Delta p_{SB}/\Delta p_{SAW}$) as a function of 1) the cardiac output CO, 2) outlet/inlet radii ratio R_{OUTLET}/R_{INLET}, 3) spatial resolution dx and 4) velocity shape coefficient β. Note the magnitude of the bias by the SB (a value larger than 1) is only affected by the shape of the velocity profile (β), and is the smallest for the bluntest profile ($\beta = 10$).

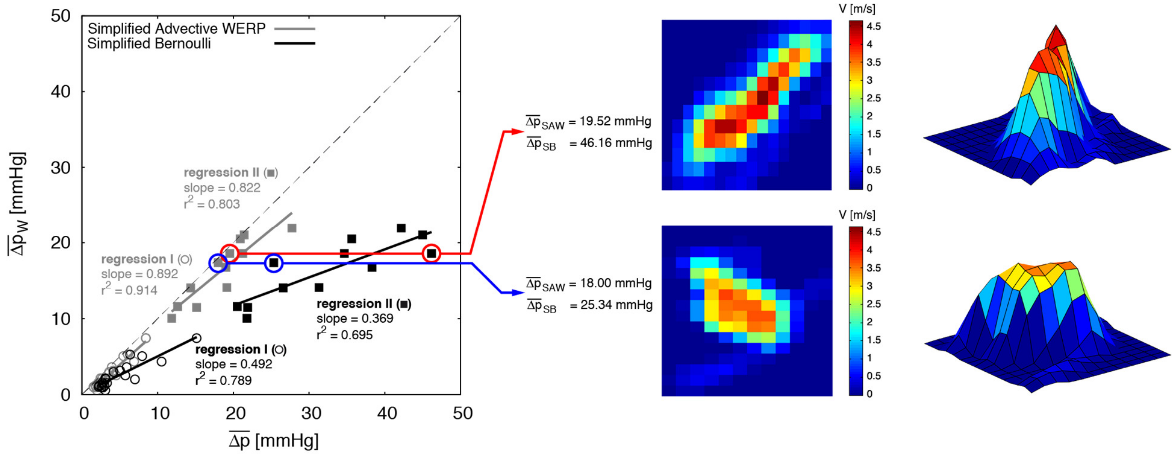

Figure A5 - Comparison of SB and SAW assessments of the pressure drop in two selected cases having similar pressure drops $\overline{\Delta p_W}$ (red and blue circles). Note how the correct computation of the advective pressure drop (using the SAW method) leads to very similar values in the two cases ($\overline{\Delta p_{SAW}}$ of 18.00 vs. 19.52 mmHg), whereas the assessment of AS severity by SB simplification (where the profile is simplified to a single velocity value) introduces a spurious difference between these two cases ($\overline{\Delta p_{SB}}$ of 25.34 vs. 46.16 mmHg).

E. Study of idealized echocardiographic velocity profiles

This section reports on the adaptation of WERP formulations to echocardiographic idealized data. To this end, the TPD obtained using the SAW formulation from the original 4D flow CMR velocity fields (Δp_{SAW}) are compared against those computed using simulated and idealized 3D Doppler (Δp_{SAW}^{D3D}) and 2D Doppler (Δp_{SAW}^{D2D}) echocardiographic data. Additionally, we jointly report TPD estimates from the SB approach (Δp_{SAW}^{D1D}).

Echocardiographic data is simulated in Plane 2 for the computation of the TPD (see Fig.1 in main manuscript). Initially, the original 4D flow CMR data is linearly interpolated onto a grid of 1 mm x 1 mm sample points over the plane. 3D Doppler echocardiographic data are defined by projection of the velocity along the direction of insonation, taking into account the funneling effect of the probe. To achieve this, the probe location is simulated 10 cm upstream of the VC in the direction of the aortic jet flow[1], thus defining an idealized 2D velocity profile by color Doppler. Similarly, a set of 1D velocity profiles from 2D color Doppler acquisition is defined by the intersection of the previously projected velocity field with hypothetical insonation planes - since velocity profiles are non-axisymmetric, a total of 12 profiles containing the peak velocity and with arbitrarily oriented lines (with increments of 15º) are generated in each case (see Supplemental material C for an illustration of the 2D and 1D profiles obtained in each case). Finally, continuous (1D) Doppler echocardiographic data is simulated using the magnitude of the peak velocity pixel projected in the aortic jet flow direction at each time point, as reported in the main manuscript.

Figure A6 illustrates the overestimation obtained with the SB formulation (regression slope of 0.522) and with the SAW formulation applied to 1D velocity profiles obtained from the simulated 2D Doppler acquisition (regression slope of 0.782 when comparing against averaging results from 12 velocity profiles in each case). The variability shown here is introduced by the arbitrary choice of the profile, determined by the insonation plane. A much larger correlation is achieved with an idealized color Doppler 3D acquisition using the SAW formulation (regression slope of 1.052).

---

[1] The aortic valve jet direction is defined as the direction of the velocity vector at the pixel with maximum velocity magnitude.

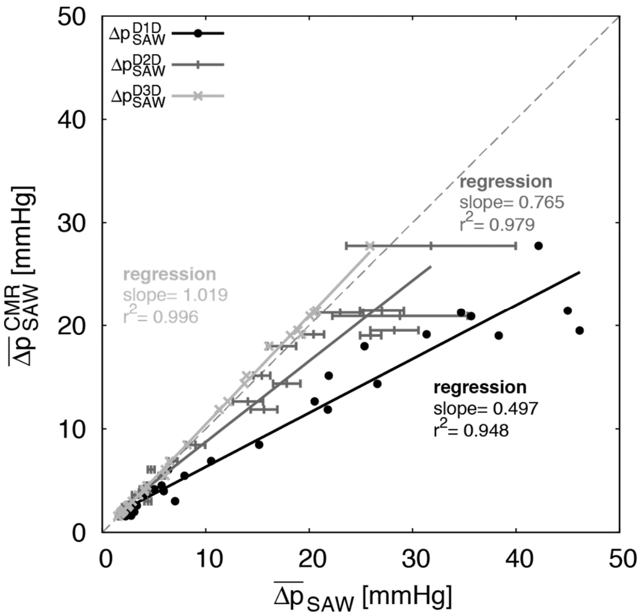

Figure A6 - Linear regression and correlation factors between mean simplified advective WERP pressure drops based on 4D flow CMR and idealized echocardiographic data: continuous wave Doppler using peak velocity values (D1D, solid black line, using Bernoulli's formulation), spatially resolved color Doppler velocity along one line of the jet cross section, as ideally obtained by a 2D ultrasound probe (D2D, dark grey line, with error bars corresponding to the range of values by the 12 orientations used to sample the complete velocity profile), and spatially resolved color Doppler velocity along in the complete cross section of the blood jet, as ideally obtained by a 3D ultrasound probe (D3D, light grey solid line). Case-specific values are reported for each Doppler based acquisition technique. Dashed grey line represents the identity.

References

1. Donati F, Figueroa CA, Smith NP, Lamata P, Nordsletten DA. Non-Invasive Pressure Difference Estimation from PC-MRI Using the Work-Energy Equation. Med Image Anal. 2015. doi:10.1016/j.media.2015.08.012.

2. Marriott M. Civil Engineering Hydraulics. John Wiley & Sons; 2009.
